# SARS-CoV-2 nucleocapsid protein triggers hyperinflammation via protein-protein interaction-mediated intracellular Cl^−^ accumulation in respiratory epithelium

**DOI:** 10.1038/s41392-022-01048-1

**Published:** 2022-07-27

**Authors:** Lei Chen, Wei-Jie Guan, Zhuo-Er Qiu, Jian-Bang Xu, Xu Bai, Xiao-Chun Hou, Jing Sun, Su Qu, Ze-Xin Huang, Tian-Lun Lei, Zi-Yang Huang, Jincun Zhao, Yun-Xin Zhu, Ke-Nan Ye, Zhao-Rong Lun, Wen-Liang Zhou, Nan-Shan Zhong, Yi-Lin Zhang

**Affiliations:** 1 School of Life Sciences, Sun Yat-sen University, Guangzhou, China; 2 State Key Laboratory of Respiratory Disease, National Clinical Research Center for Respiratory Disease, Guangzhou Institute of Respiratory Health, The First Affiliated Hospital of Guangzhou Medical University, Guangzhou Medical University, Guangzhou, China; 3 Department of Thoracic Surgery, Guangzhou Institute for Respiratory Health, The First Affiliated Hospital of Guangzhou Medical University, Guangzhou Medical University, Guangzhou, China; 4 Guangzhou Laboratory, Guangzhou, China

**Keywords:** Physiology, Respiratory tract diseases

## Abstract

SARS-CoV-2, the culprit pathogen of COVID-19, elicits prominent immune responses and cytokine storms. Intracellular Cl^−^ is a crucial regulator of host defense, whereas the role of Cl^−^ signaling pathway in modulating pulmonary inflammation associated with SARS-CoV-2 infection remains unclear. By using human respiratory epithelial cell lines, primary cultured human airway epithelial cells, and murine models of viral structural protein stimulation and SARS-CoV-2 direct challenge, we demonstrated that SARS-CoV-2 nucleocapsid (N) protein could interact with Smad3, which downregulated cystic fibrosis transmembrane conductance regulator (CFTR) expression via microRNA-145. The intracellular Cl^−^ concentration ([Cl^−^]_i_) was raised, resulting in phosphorylation of serum glucocorticoid regulated kinase 1 (SGK1) and robust inflammatory responses. Inhibition or knockout of SGK1 abrogated the N protein-elicited airway inflammation. Moreover, N protein promoted a sustained elevation of [Cl^−^]_i_ by depleting intracellular cAMP via upregulation of phosphodiesterase 4 (PDE4). Rolipram, a selective PDE4 inhibitor, countered airway inflammation by reducing [Cl^−^]_i_. Our findings suggested that Cl^−^ acted as the crucial pathological second messenger mediating the inflammatory responses after SARS-CoV-2 infection. Targeting the Cl^−^ signaling pathway might be a novel therapeutic strategy for COVID-19.

## Introduction

Coronavirus disease 2019 (COVID-19) was caused by the infection of severe acute respiratory syndrome coronavirus 2 (SARS-CoV-2). SARS-CoV-2 is a member of β-coronavirus with a single-strand RNA genome which bears ~80 and 50% identity as compared with that of SARS-CoV and Middle East respiratory syndrome coronavirus (MERS-CoV), respectively.^1^ The SARS-CoV-2 genome encodes 27 proteins including the spike (S) and nucleocapsid (N) proteins, which could interact with various host proteins.^2^ Despite the efforts to curb further spreading, there remain few effective targeted drugs for COVID-19. This highlights the urgent need to elucidate the pathogenesis of SARS-CoV-2 by exploring how the viral proteins elicit pulmonary inflammatory responses.

The respiratory epithelium regulates water and ion transport, serving as the first-line structural barrier of the host defense system against pathogens.^3,4^ Viruses could trigger innate immune responses within the respiratory epithelial cells (RECs), leading to epithelial apoptosis, prominent inflammatory responses, mucus hypersecretion, and secondary microbial infections.^5^ Upon SARS-CoV-2 infection, RECs could release multiple pro-inflammatory mediators.^6^ However, the mechanisms underlying the respiratory epithelium-mediated host defense responses during SARS-CoV-2 infection remain largely unclear.

Cl^−^ plays a crucial role in regulating cellular functions such as ion homeostasis, cell proliferation and differentiation, mucus production, oxidative stress, and innate immunity.^7,8,9^ Within the RECs, the intracellular Cl^−^ concentration ([Cl^−^]_i_) is regulated by ion transport proteins including the cystic fibrosis transmembrane conductance regulator (CFTR), a major cAMP-dependent anion channel responsible for transepithelial Cl^−^ transport.^7^ CFTR reportedly regulated the host defense against pathogen infection, whereas functional deficiencies and downregulation of CFTR resulted in defective host defenses.^10^ For instance, cystic fibrosis (a disease characterized by CFTR monogenic mutations) manifests as airway mucus retention, microbial infections, and bronchiectasis that have been associated with chronic neutrophilic inflammation.^11^ Additionally, the H1N1 virus frequently elicited infections among patients with cystic fibrosis, resulting in pulmonary exacerbations which were characterized by more aggravated respiratory symptoms, a greater risk of mechanical ventilation use and death^12^. These observations indicated that abnormal CFTR function might be a key factor predisposing to the development of severe pneumonia or recalcitrant airway microbial infections, and therefore, CFTR has been hypothesized as a therapeutic target of COVID-19.^13^ Here, we stimulated the RECs with the SARS-CoV-2 structural proteins to investigate whether the CFTR-Cl^−^ signaling pathway is implicated in exuberant airway inflammation caused by SARS-CoV-2 infection, and further elucidate the underlying mechanisms.

## Results

### SARS-CoV-2 N protein-elicited inflammatory responses within the RECs

**Fig. 1:**
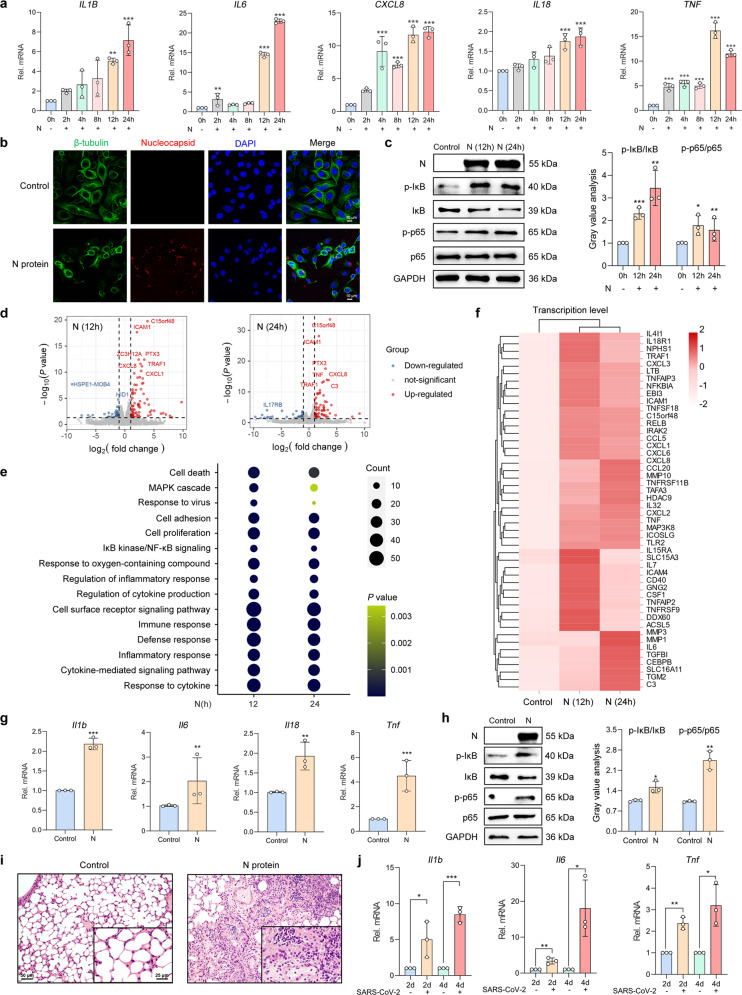
SARS-CoV-2 N protein-elicited respiratory epithelial inflammation. **a** The mRNA expression levels of pro-inflammatory cytokines in N protein (50 μg/ml)-stimulated BEAS-2B cells (*n* = 3). **b** Fluorescence labeling of β-tubulin (green) and N protein (red) in BEAS-2B cells with DAPI-labeled nuclei (blue) after stimulation with N protein (50 μg/ml) for 24 h. Scale bars, 10 μm. **c** The phosphorylation level of IκB and NF-κB p65 subunit after N protein (50 μg/ml) stimulation in BEAS-2B cells, with the gray value analysis (*n* = 3). **d** Volcano plot visualization of the global RNA-seq DEGs in N protein (50 μg/ml)-stimulated BEAS-2B cells. **e** Dot plot of the enriched GO terms of the mRNAs exhibiting the enriched genes of BEAS-2B cells which were stimulated with N protein (50 μg/ml). **f** A heatmap showing the mRNA expression levels of the DEGs upregulated by N protein (50 μg/ml) stimulation compared with the control group, with the genes belonging to the GO annotations for cytokine activity and chemokine activity (GO: 0005125 and GO: 0008009). **g** The mRNA expression levels of cytokines in mice stimulated with N protein (0.25 mg/kg) (*n* = 3). **h** The phosphorylation level of IκB and p65 after N protein (0.25 mg/kg) stimulation in mice, with the gray value analysis (*n* = 3). **i** H&E staining of lung slices from N protein (0.25 mg/kg)-stimulated mice. Scale bars, 50 and 25 μm. **j** The mRNA expression levels of cytokines in SARS-CoV-2 (1 × 10^5^ PFU)-infected hACE2-transduced mice (*n* = 3). Data were shown as mean ± SD, ^*^P < 0.05, ^**^P < 0.01, ^***^P < 0.001 compared with the control group or indicated by lines

Pneumonia has been a common pulmonary manifestation in patients with COVID-19. Moreover, severe COVID-19 patients reportedly had prominent inflammatory responses, characterized by the markedly raised concentration of pro-inflammatory cytokines.^14^ Therefore, we initially determined the expression of pro-inflammatory cytokines in RECs after stimulation with the SARS-CoV-2 structural proteins (S1, S2, and N proteins). Interestingly, stimulating the human REC cell lines BEAS-2B and Calu-3 cells with either S1 or S2 proteins (50 μg/ml) failed to affect the expression of cytokines (Supplementary Fig. S1a, b). However, N protein (50 μg/ml) stimulation consistently upregulated cytokine mRNA expression in BEAS-2B, A549, and Calu-3 cells (Fig. 1a and Supplementary Fig. S2a, b). We next visualized N protein within the RECs using a confocal imaging system, revealing the localization within the cytoplasm and a minority of the nuclei of BEAS-2B cells (Fig. 1b), which indicated the endocytosis of N proteins. NF-κB is a critical regulator modulating airway inflammation in infectious diseases.^15^ After N protein stimulation, both IκB and NF-κB p65 subunit were phosphorylated (Fig. 1c), implying the activation of NF-κB signaling in RECs. To reveal the transcriptional landscape in response to N protein stimulation, we further performed RNA sequencing (RNA-seq) analysis. The volcano plot revealed the differentially expressed genes (DEGs) after N protein stimulation in BEAS-2B cells (Fig. 1d). The gene ontology (GO) enrichment analyses illustrated upregulation of the “response to cytokine”, “inflammatory response”, and “IκB/NF-κB signaling” modules (Fig. 1e). The heatmap of DEGs correlated with the mRNA expression levels of *IL6*, *IL7*, *CXCL6*, *CXCL8*, *CCL5, TNF*, etc. (Fig. 1f). These findings demonstrated that N protein stimulation triggered inflammatory responses in the RECs.

Next, we evaluated the effect of N protein stimulation on airway inflammation in mice via intratracheal instillation of the N protein. Consistent with the observations in vitro, the expression of the pro-inflammatory cytokines was upregulated in the mice lungs (Fig. 1g). Additionally, we have documented pronounced IκB as well as p65 phosphorylation (Fig. 1h), and prominent immune cell infiltration (Fig. 1i) within the lungs of N protein-stimulated mice. We also evaluated the systemic inflammatory responses in this model. Interestingly, upregulated cytokine expression and immune cell infiltration were observed in both the blood and important organs (eg. heart, liver, and kidney) of the mice (Supplementary Fig. S3), which indicated that intratracheal instillation of the N protein caused extensive damage in mice. Finally, we established a SARS-CoV-2 direct challenge murine model for assessing pulmonary pathology. The mice transduced with the human angiotensin-converting enzyme 2 (hACE2) were infected with SARS-CoV-2 (1 × 10^5^ PFU), resulting in robust pathological changes associated with pneumonia (Fig. 1j and Supplementary Fig. S4). Overall, both in vitro and in vivo results suggested that SARS-CoV-2 N protein, but not S protein, elicited NF-κB-mediated inflammatory responses within the RECs.

### N protein downregulated CFTR expression and increased [Cl^−^]_i_ in RECs

**Fig. 2:**
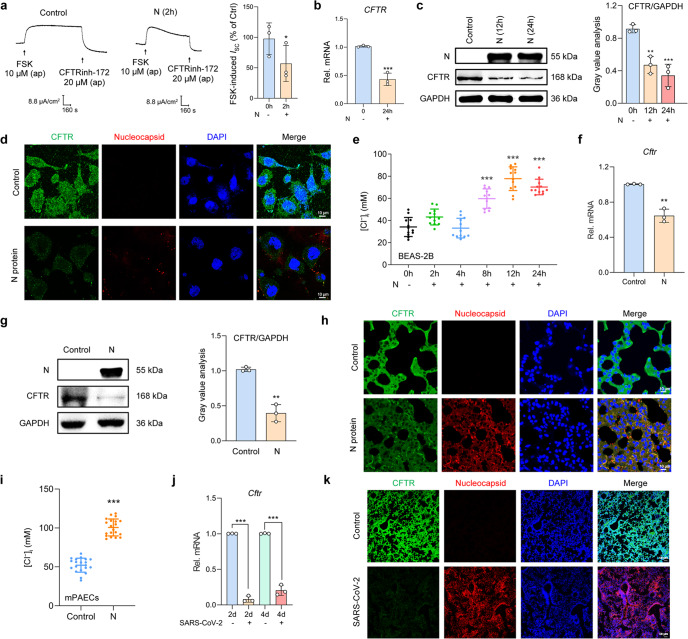
SARS-CoV-2 N protein triggered the downregulation of CFTR and increased [Cl^−^]_i_ in RECs. **a** Representative traces showing the effect of N protein (50 μg/ml) on *I*_SC_ responses induced by forskolin (FSK, 10 μM) in 16HBE14o- cells, with the statistical analysis (*n* = 3). **b** The mRNA expression levels of *CFTR* in N protein (50 μg/ml)-stimulated BEAS-2B cells (*n* = 3). **c** The expression of CFTR after N protein (50 μg/ml) stimulation in BEAS-2B cells, with the gray value analysis (*n* = 3). **d** Fluorescence labeling of CFTR (green) and N protein (red) in BEAS-2B cells with DAPI-labeled nuclei (blue) after stimulation with N protein (50 μg/ml) for 24 h. Scale bars, 10 μm. **e** The changes in [Cl^−^]_i_ of BEAS-2B cells after N protein (50 μg/ml) stimulation (*n* = 12 cells). **f** The mRNA expression level of *Cftr* in the lungs of N protein (0.25 mg/kg)-stimulated mice (*n* = 3). **g** The expression of CFTR after N protein (0.25 mg/kg) stimulation in the lungs of mice, with the gray value analysis (*n* = 3). **h** Fluorescence labeling of CFTR (green) and N protein (red) in mouse lung slices with DAPI-labeled nuclei (blue) after N protein (0.25 mg/kg) stimulation. Scale bars, 10 μm. **i** The changes in [Cl^−^]_i_ of mPAECs from mice after N protein (0.25 mg/kg) stimulation (*n* = 20 cells). **j** The mRNA expression level of *Cftr* in the lungs of hACE2-transduced mice infected with SARS-CoV-2 (1 × 10^5^ PFU) (*n* = 3). **k** Fluorescence labeling of CFTR (green) and N protein (red) in lung slices of hACE2-transduced mice infected with SARS-CoV-2 (1 × 10^5^ PFU). Scale bars, 50 μm. Data were shown as mean ± SD, ^*^P < 0.05, ^**^P < 0.01, ^***^P < 0.001 compared with the control group or indicated by lines

Considering the association between Cl^−^ channel disorders and defective host defense, we next examined the CFTR-mediated transepithelial Cl^−^ secretion after N protein stimulation in RECs. The short-circuit current (*I*_SC_) measurement revealed the impaired transepithelial Cl^−^ secretion mediated by CFTR in the N protein-stimulated 16HBE14o- cells (Fig. 2a). In addition, quantitative real-time PCR (qPCR), Western blot, and immunofluorescence analysis have consistently demonstrated downregulated CFTR expression after N protein stimulation in BEAS-2B cells (Fig. 2b–d). Given that CFTR regulates intracellular Cl^−^ signaling in epithelial cells,^16^ we further detected the changes in [Cl^−^]_i_ of N protein-stimulated BEAS-2B cells. Notably, the [Cl^−^]_i_ increased from 34.16 ± 2.49 mM to 77.77 ± 3.09 mM (Fig. 2e) after N protein stimulation for 12 h, confirming the disequilibrium in [Cl^−^]_i_ of RECs. We next analyzed whether these effects could also be elicited by other structural proteins. Consistent with the findings from the inflammatory marker assays, neither the S1 nor S2 proteins could markedly affect the transepithelial Cl^−^ secretion or [Cl^−^]_i_ (Supplementary Fig. S1c, d). These results indicated that N protein, but not S protein, contributed to the heightened [Cl^−^]_i_ in RECs.

We next performed in vivo studies to further validate the above-mentioned findings. CFTR expression was downregulated within the RECs in mice intratracheally instilled with N protein (Fig. 2f–h and Supplementary Fig. S2d) or infected with SARS-CoV-2 (Fig. 2j, k). In addition, [Cl^−^]_i_ was significantly elevated in the primary cultured airway epithelial cells isolated from the N protein-stimulated mice (mPAECs) (Fig. 2i). All these findings further confirmed the defective CFTR-Cl^−^ signaling in RECs infected with SARS-CoV-2.

### N protein interacted with Smad3 to trigger the downregulation of CFTR and elevation in [Cl^−^]_i_ via microRNA-145 (miR-145)

**Fig. 3:**
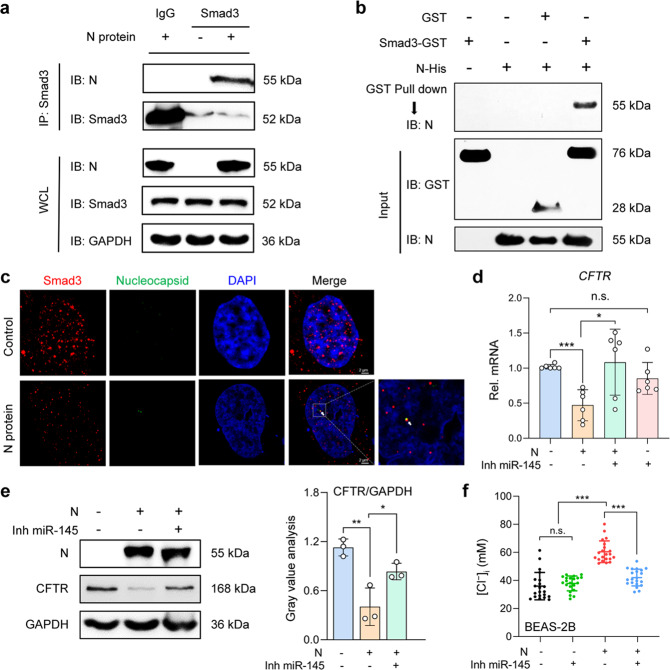
SARS-CoV-2 N protein interacted with Smad3 to induce miR-145-mediated downregulation of CFTR and increased [Cl^−^]_i_ in RECs. **a** Whole-cell lysates (WCL) from BEAS-2B cells stimulated by N protein (50 μg/ml) for 24 h were subject to immunoprecipitation with anti-Smad3 beads and immunoblotted with an anti-N protein antibody. **b** GST pull-down assays using GST-Smad3 and N protein. N proteins binding to GST-Smad3 were detected by using an anti-N protein antibody. **c** Fluorescence labeling of Smad3 (red) and N protein (green) in BEAS-2B cells with DAPI-labeled nuclei (blue) after stimulation with N protein (50 μg/ml) for 24 h. Scale bars, 2 μm. **d** The effect of Inh miR-145 (100 nM) on the mRNA expression levels of *CFTR* in BEAS-2B cells stimulated with N protein (50 μg/ml) for 24 h (*n* = 3). **e** The effect of Inh miR-145 (100 nM) on the expression of CFTR in BEAS-2B cells stimulated by N protein (50 μg/ml) for 24 h, with the gray value analysis (*n* = 3). **f** The effect of Inh miR-145 (100 nM) on [Cl^−^]_i_ of BEAS-2B cells stimulated by N protein (50 μg/ml) for 24 h (*n* = 20 cells). Data are shown as mean ± SD, ^*^P < 0.05, ^**^P < 0.01, ^***^P < 0.001, n.s. *P* > 0.05

We next investigated the mechanisms underlying the N protein-induced downregulation of CFTR in RECs. Transforming growth factor-1 (TGF-β1) reportedly activated Smad3, which triggered CFTR downregulation via augmenting miR-145 expression.^17–20^ More importantly, microarray data showed that miR-145 was one of the five highly expressed miRNAs and small nucleolar RNA in patients with severe COVID-19.^21^ Given that numerous Smad-interacting proteins have been implicated in Smad signaling,^22^ we postulated that N protein might interact with Smad3 to induce the downregulation of CFTR via miR-145. The co-immunoprecipitation results revealed the binding of N protein with Smad3 in BEAS-2B cells under physiological conditions (Fig. 3a). Additionally, the GST pull-down assessment showed an efficient pull-down of the N protein by the GST-tagged Smad3 protein, while the binding of N protein to GST alone was not observed (Fig. 3b). We also performed immunofluorescence assays, revealing the co-localization of N protein and Smad3 within the nuclei of BEAS-2B cells (Fig. 3c). These results confirmed that N protein could directly bind to Smad3 and that the N protein-Smad3 complex could enter into the nuclei of RECs.

We then validated the involvement of miR-145 in N protein-induced downregulation of CFTR and elevation in [Cl^−^]_i_. The inhibitor of miR-145 (Inh miR-145, 100 nM) restored CFTR expression in N protein-stimulated BEAS-2B cells (Fig. 3d, e). Moreover, Inh miR-145 decreased [Cl^−^]_i_ to the normal physiological level after N protein stimulation (Fig. 3f). Taken together, SARS-CoV-2 N protein could interact with Smad3, promoting the downregulation of CFTR and an increase of [Cl^−^]_i_ via miR-145 in RECs.

### N protein aggravated the inflammation via the Cl^−^-sensing serum glucocorticoid regulated kinase 1 (SGK1)

**Fig. 4:**
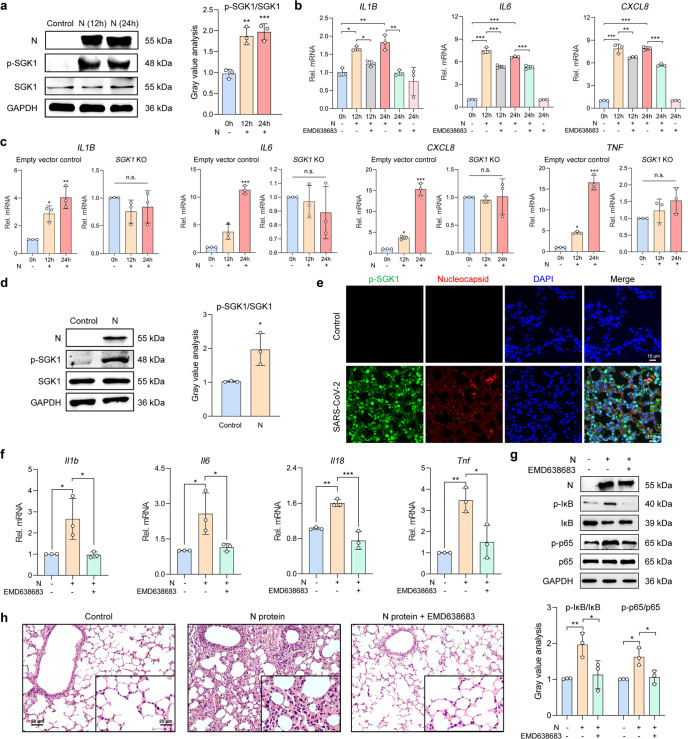
SARS-CoV-2 N protein-triggered inflammation via the activation of SGK1 in RECs. **a** The phosphorylation level of SGK1 after N protein (50 μg/ml) stimulation in BEAS-2B cells, with the gray value analysis (*n* = 3). **b** The effect of EMD638683 (50 μM) on the mRNA expressions of pro-inflammatory cytokines in N protein (50 μg/ml)-stimulated BEAS-2B cells (*n* = 3). **c** The mRNA expressions of cytokines after N protein (50 μg/ml) stimulation in the empty vector control or *SGK1* KO BEAS-2B cells (*n* = 3). **d** The phosphorylation level of SGK1 in lungs of N protein (0.25 mg/kg)-stimulated mice, with the gray value analysis (*n* = 3). **e** Fluorescence labeling of phosphorylated SGK1 (green) and N protein (red) in lung slices of hACE2-transduced mice infected with SARS-CoV-2 (1 × 10^5^ PFU). Scale bars, 10 μm. **f** The effect of EMD638683 (10 mg/kg) on the mRNA expressions of cytokines in N protein (0.25 mg/kg)-stimulated mice (*n* = 3). **g** The effect of EMD638683 (10 mg/kg) on the phosphorylation level of IκB and NF-κB p65 subunit in N protein (0.25 mg/kg)-stimulated mice, with the gray value analysis (*n* = 3). **h** H&E staining of lung slices from N protein (0.25 mg/kg)-stimulated mice with or without treatment with EMD638683 (10 mg/kg). Scale bars, 50 μm and 25 μm. Data were shown as mean ± SD, ^*^P < 0.05, ^**^P < 0.01, ^***^P < 0.001 compared with the control group or indicated by lines, n.s. *P* > 0.05

SGK1 was a Cl^−^-sensing kinase, which mediated the inflammatory response in RECs during bacterial infections, including in patients with bronchiectasis.^23^ Because [Cl^−^]_i_ was elevated after N protein stimulation, we next investigated whether SGK1 was implicated in the N protein-induced epithelial inflammatory responses. Phosphorylation of SGK1 was augmented after N protein stimulation, whereas the total SGK1 expression remained constant (Fig. 4a). Pretreatment with EMD638683 (50 μM), a selective SGK1 inhibitor, suppressed pro-inflammatory cytokine expression in BEAS-2B cells (Fig. 4b and Supplementary Fig. S5a). To further validate these findings, we established a CRISPR-Cas9-based *SGK1* knockout (KO) REC model. Compared with the empty vector control, N protein did not alter cytokine expression in the *SGK1* KO cells (Fig. 4c and Supplementary Fig. S5b), suggesting that SGK1 was indispensable in modulating the N protein-induced ongoing inflammation in RECs.

We also investigated the involvement of SGK1 in N protein-induced pneumonia in vivo. The phosphorylation of SGK1 was augmented in the lungs of the N protein-stimulated (Fig. 4d) or SARS-CoV-2-challenged (Fig. 4e) mice. In addition, pretreatment with EMD638683 (10 mg/kg) significantly attenuated the upregulation of cytokines (Fig. 4f) and activation of NF-κB (Fig. 4g) after N protein stimulation in mice. Histopathological analysis displayed that EMD638683 mitigated alveoli destruction, immune cell infiltration, and exudation in the N protein-stimulated mice (Fig. 4h). These data implied that SARS-CoV-2 N protein aggravated the airway inflammation via the Cl^−^-sensing SGK1 in RECs.

### The increased [Cl^−^]_i_ was sustained by N protein stimulation via NF-κB-phosphodiesterase 4 (PDE4)-cAMP signaling pathways

**Fig. 5:**
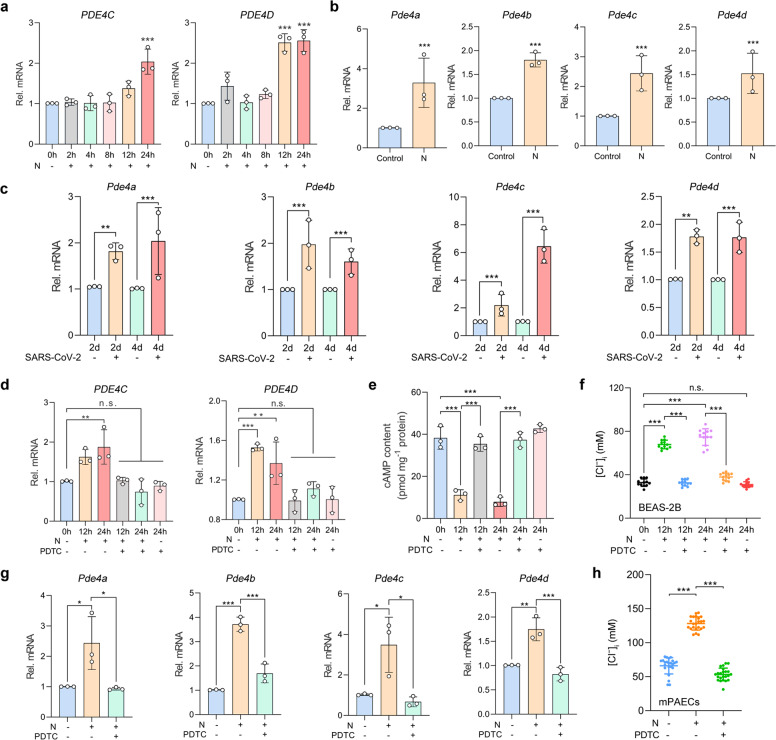
SARS-CoV-2 N protein contributed to the sustained elevation in [Cl^−^]_i_ via NF-κB-PDE4-cAMP signaling pathway in RECs. **a** The mRNA expressions of *PDE4C* and *PDE4D* in the N protein (50 μg/ml)-stimulated BEAS-2B cells (*n* = 3). **b** The mRNA expressions of *Pde4* in the lung tissues of N protein (0.25 mg/kg)-stimulated mice (*n* = 3). **c** The mRNA expressions of *Pde4* in the lung tissues of SARS-CoV-2 (1 × 10^5^ PFU)-infected hACE2-transduced mice (*n* = 3). **d** The effect of PDTC (100 μM) on *PDE4* mRNA expression in BEAS-2B cells stimulated with N protein (50 μg/ml) (*n* = 3). **e** The effect of PDTC (100 μM) on intracellular cAMP levels in BEAS-2B cells stimulated with N protein (50 μg/ml) (*n* = 3). **f** The effect of PDTC (100 μM) on [Cl^−^]_i_ of BEAS-2B cells stimulated with the N protein (50 μg/ml) (*n* = 12 cells). **g** The effect of PDTC (100 mg/kg) on *Pde4* mRNA expression in the lung tissues of N protein (0.25 mg/kg)-stimulated mice (*n* = 3). **h** The effect of PDTC (100 mg/kg) on [Cl^−^]_i_ of mPAECs from mice after N protein (0.25 mg/kg) stimulation (*n* = 20 cells). Data were shown as mean ± SD, ^*^P < 0.05, ^**^P < 0.01, ^***^P < 0.001 compared with the control group or indicated by lines, n.s. *P* > 0.05

The activity of CFTR was regulated by the intracellular cAMP concentration.^24^ We have previously demonstrated that PDE4 (which degraded intracellular cAMP) could modulate [Cl^−^]_i_ and promote the ongoing epithelial inflammation during bacterial infections.^23^ Here, we have further shown that the mRNA expression levels of *PDE4C* and *PDE4D* were upregulated at 12 h and thereafter following N protein stimulation in BEAS-2B cells (Fig. 5a and Supplementary Fig. S6). Notably, the mRNA of four subtypes of PDE4 were consistently upregulated in the lungs of mice intratracheally instilled with the N protein or challenged with SARS-CoV-2 (Fig. 5b, c).

We next clarified the mechanisms underlying the upregulation of PDE4 and heightened [Cl^−^]_i_ after N protein stimulation. Pretreatment with PDTC (100 μM), an inhibitor of NF-κB,^25^ suppressed the upregulation of *PDE4C* and *PDE4D* mRNA expression and decreased intracellular cAMP level induced by N protein within BEAS-2B cells (Fig. 5d, e). Moreover, PDTC also inhibited the elevation in [Cl^−^]_i_ in N protein-stimulated BEAS-2B cells (Fig. 5f). Similarly, PDTC (100 mg/kg) significantly reversed the upregulation of PDE4 subtypes (Fig. 5g) and the elevation of [Cl^−^]_i_ of mPAECs (Fig. 5h) caused by N protein stimulation in mice. These results indicated that SARS-CoV-2 N protein induced the upregulation of PDE4 expression through activation of NF-κB signaling pathways, thus leading to the sustained elevation in [Cl^−^]_i_ of RECs.

### Inhibition of PDE4 suppressed the N protein-induced respiratory epithelial hyperinflammation via reduction of the [Cl^−^]_i_

**Fig. 6:**
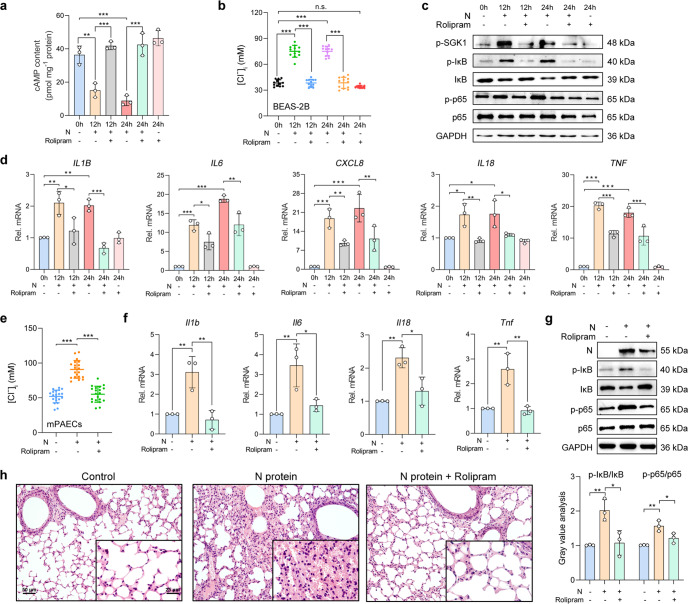
Inhibition of PDE4 suppressed the SARS-CoV-2 N protein-induced ongoing inflammation via downregulation of the [Cl^−^]_i_. **a** The effect of rolipram (20 μM) on the intracellular cAMP content after the N protein (50 μg/ml) stimulation in BEAS-2B cells (*n* = 3). **b** The effect of rolipram (20 μM) on [Cl^−^]_i_ in BEAS-2B cells stimulated with the N protein (50 μg/ml) (*n* = 12 cells). **c** The effect of rolipram (20 μM) on phosphorylation level of SGK1, IκB, and NF-κB p65 subunit after N protein (50 μg/ml) stimulation in BEAS-2B cells. **d** The effect of rolipram (20 μM) on the mRNA expression levels of pro-inflammatory cytokines stimulated with the N protein (50 μg/ml) in BEAS-2B cells (*n* = 3). **e** The effect of rolipram (10 mg/kg) on [Cl^−^]_i_ in mPAECs from mice after N protein (0.25 mg/kg) stimulation (*n* = 20 cells). **f** The effect of rolipram (10 mg/kg) on the expression levels of cytokines stimulated with the N protein (0.25 mg/kg) in mice (*n* = 3). **g** The effect of rolipram (10 mg/kg) on the phosphorylation level of IκB and p65 after N protein (0.25 mg/kg) stimulation in mice, with the gray value analysis (*n* = 3). **h** H&E staining of lung slices from N protein (0.25 mg/kg)-stimulated mice with or without treatment with rolipram (10 mg/kg). Scale bars, 50 μm and 25 μm. Data were shown as mean ± SD, ^*^P < 0.05, ^**^P < 0.01, ^***^P < 0.001, n.s. *P* > 0.05

Because N protein induced the upregulation of PDE4 expression, which led to the sustained elevation in [Cl^−^]_i_, we sought to examine whether inhibition of PDE4 could attenuate intracellular Cl^−^ accumulation and airway inflammation induced by N protein. Pretreatment with rolipram (20 μM), a selective inhibitor of PDE4,^26^ effectively reversed the effect of N protein on intracellular cAMP concentration (Fig. 6a) and [Cl^−^]_i_ (Fig. 6b) in BEAS-2B cells. Additionally, rolipram abolished the enhanced phosphorylation of SGK1, IκB as well as p65 (Fig. 6c), and the upregulation of pro-inflammatory cytokine expression (Fig. 6d) in N protein-stimulated BEAS-2B cells.

The in vivo study also revealed that rolipram (10 mg/kg) abrogated the intracellular Cl^−^ accumulation of mPAECs from the N protein-intratracheally instilled mice (Fig. 6e). Moreover, upregulation of the cytokines (Fig. 6f), activation of NF-κB (Fig. 6g), and immune cell infiltration (Fig. 6h) were all suppressed by rolipram. Therefore, inhibition of PDE4 protected against SARS-CoV-2 N protein-induced respiratory epithelial inflammation via reduction of the [Cl^−^]_i_.

### N protein aggravated inflammation via promoting intracellular Cl^−^ accumulation mediated by N protein-Smad3 interactions in human primary cultured airway epithelial cells (hPAECs)

**Fig. 7:**
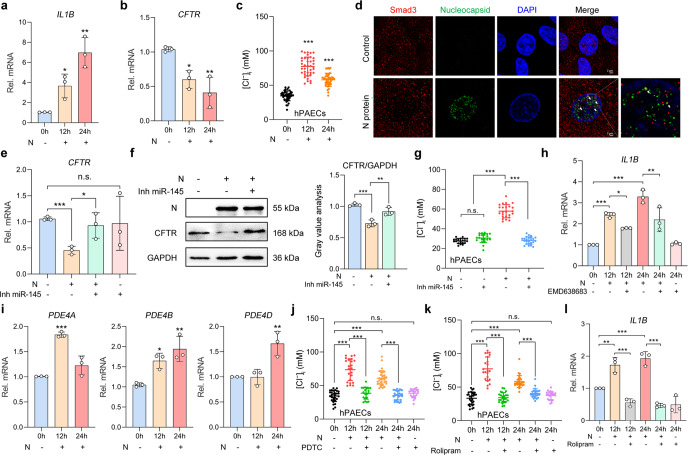
Involvement of Cl^−^ signaling mediated by SARS-CoV-2 N protein-Smad3 interactions in N protein-triggered inflammatory responses in hPAECs. **a** The mRNA expression levels of *IL1B* in N protein (50 μg/ml)-stimulated hPAECs (*n* = 3). **b** The mRNA expression levels of *CFTR* in N protein (50 μg/ml)-stimulated hPAECs (*n* = 3). **c** The changes in [Cl^−^]_i_ of hPAECs after N protein (50 μg/ml) stimulation (*n* = 39 cells from 3 individuals). **d** Fluorescence labeling of Smad3 (red) and N protein (green) in hPAECs with DAPI-labeled nuclei (blue) after stimulation with N protein (50 μg/ml) for 24 h. Scale bars, 2 μm. **e** The effect of Inh miR-145 (100 nM) on the mRNA expression levels of *CFTR* in hPAECs stimulated with N protein (50 μg/ml) for 24 h (*n* = 3). **f** The effect of Inh miR-145 (100 nM) on the expression of CFTR in hPAECs stimulated by N protein (50 μg/ml) for 24 h, with the gray value analysis (*n* = 3). **g** The effect of Inh miR-145 (100 nM) on the [Cl^−^]_i_ of hPAECs stimulated with N protein (50 μg/ml) for 24 h (*n* = 20 cells from three individuals). **h** The effect of EMD638683 (50 μM) on the mRNA expressions of *IL1B* in N protein (50 μg/ml)-stimulated hPAECs (*n* = 3). **i** The mRNA expressions of *PDE4* in the N protein (50 μg/ml)-stimulated hPAECs (*n* = 3). **j** The effect of PDTC (100 μM) on the [Cl^−^]_i_ of the N protein (50 μg/ml)-stimulated hPAECs (*n* = 20 cells from three individuals). **k** The effect of rolipram (20 μM) on [Cl^−^]_i_ of the N protein (50 μg/ml)-stimulated hPAECs (*n* = 3 cells from 3 individuals). **l** The effect of rolipram (20 μM) on the mRNA expression levels of *IL1B* in the N protein (50 μg/ml)-stimulated hPAECs (*n* = 3). Data were shown as mean ± SD, ^*^P < 0.05, ^**^P < 0.01, ^***^P < 0.001 compared with the control group or indicated by lines, n.s. *P* > 0.05

To provide a more compelling conclusion, we have verified the core findings in the hPAECs. After stimulation with N protein, robust inflammatory responses (Fig. 7a and Supplementary Fig. S7b), downregulation of CFTR (Fig. 7b), and increased [Cl^−^]_i_ (Fig. 7c) were consistently observed in hPAECs. In addition, the co-localization of N protein and Smad3 within the nuclei (Fig. 7d), and the involvement of miR-145 in CFTR downregulation (Fig. 7e, f) and elevation in [Cl^−^]_i_ (Fig. 7g) were also confirmed in N protein-stimulated hPAECs. We next verified the downstream pathways related to the intracellular Cl^−^ accumulation. Inhibition of SGK1 by pretreatment with EMD638683 (50 μM) attenuated the inflammatory responses in the N protein-stimulated hPAECs (Fig. 7h and Supplementary Fig. S7c). Interestingly, all PDE4 subtypes except for PDE4C were upregulated after N protein stimulation in hPAECs (Fig. 7i and Supplementary Fig. S7d), a finding inconsistent with what we have observed in BEAS-2B cells. Nevertheless, inhibition of either NF-κB or PDE4 alleviated the elevation in [Cl^−^]_i_ and inflammatory responses in hPAECs (Fig. 7j–l and Supplementary Fig. S7e).

## Discussion

**Fig. 8:**
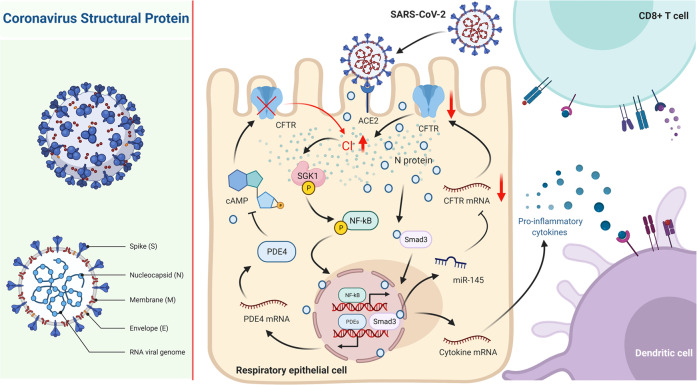
Schematic diagram depicting the role of intracellular Cl^−^ accumulation in eliciting an ongoing inflammatory response after SARS-CoV-2 N protein stimulation in RECs. In RECs, the N protein interacted with Smad3, which triggered the downregulation of the CFTR via miR-145. Thereafter, the [Cl^−^]_i_ was elevated and elicited inflammatory responses through activating the Cl^−^-sensing SGK1. Moreover, the expression of PDE4 was upregulated owing to the activation of NF-κB, which resulted in cAMP degradation and dysfunction of CFTR, contributing to the sustained high [Cl^−^]_i_ and ongoing airway inflammation. [Created with BioRender.com (Canada)]

SARS-CoV-2 has spread globally and caused pneumonia which is associated with exuberant inflammatory responses. Here, we have demonstrated that SARS-CoV-2 N protein, but not S proteins (S1 and S2), could directly bind to the Smad3 protein and triggered the downregulation of CFTR via miR-145, which then induced an elevation of [Cl^−^]_i_ and inflammation through activating the Cl^−^-sensing protein SGK1 in RECs. Moreover, activation of NF-κB augmented PDE4 expression, which resulted in cAMP degradation and sustained CFTR dysfunction, contributing to the persistently increased [Cl^−^]_i_ and ongoing airway inflammation (Fig. 8).

As the core structural protein of SARS-CoV-2 responsible for the binding with host ACE2 to facilitate cell entry, S protein could also interact with bacterial lipopolysaccharide and aggravate inflammation.^27^ In our study, stimulation with neither S1 nor S2 protein could trigger prominent inflammatory responses within the RECs, which was inconsistent with the observations in macrophages.^28–30^ Nevertheless, we and others have collectively confirmed that N protein plays a crucial role in eliciting augmented immune responses in both RECs and macrophages.^31,32^ The difference in the magnitude of inflammatory responses elicited by SARS-CoV-2 structural protein stimulation could be interpreted by the absence of the corresponding pattern recognition receptors in the RECs because the S protein of both SARS-CoV and SARS-CoV-2 could induce marked inflammatory responses in the immune cells as described previously.^33^

CFTR has been shown to be a crucial regulator of microbial infection, mucus obstruction, and airway inflammation.^34,35^ Here, we have demonstrated that CFTR expression was markedly downregulated within both the N protein-stimulated RECs and SARS-CoV-2-infected mice. The virulent influenza viruses could induce the downregulation of CFTR in alveolar epithelial cells, leading to failure of alveolar fluid clearance and protein permeability.^10^ Furthermore, defective CFTR expression has been observed in influenza virus M2-infected bronchial epithelial cells and respiratory syncytial virus-infected mice.^36^ These findings consistently suggested that the expression of CFTR negatively correlated with respiratory virus infection, but the underlying mechanism has not been clarified. TGF-β1, which was released during SARS-CoV-2 infection, reportedly decreased CFTR mRNA expression through activating the Smad2/3 receptor and the recruitment of miR-143 and miR-145 to the RNA-induced silencing complex in human airway epithelial cells.^18,20,37^ Here, we have further demonstrated that N protein could directly interact with Smad3 and recruited miR-145 to downregulate the expression of CFTR. Our findings have significantly extended the regulatory mechanism underlying the pathogen-induced downregulation of CFTR which is not solely associated with aberrant TGF-β signaling as described previously.^37^ On the other hand, a high concentration (>1 ng/ml) of IL-1β reportedly downregulated CFTR mRNA expression in T84 cells by modulating the NF-κB pathway. In the light of the markedly elevated IL-1β concentration (>1 ng/ml) in cells infected with SARS-CoV-2,^38^ the N-protein-induced defective CFTR expression might also be mediated by the IL-1β-NF-κB signaling pathways. Additionally, previous evidence revealed that the N protein of the porcine enteric coronavirus could interact with specific gene promoters, thereby affecting the gene expressions.^39^ Thus, in-depth studies could be conducted to investigate the direct interactions between N protein and the CFTR gene promoter.

The role of Cl^−^ in regulating cellular physiological functions such as cell volume has been well documented.^8^ Under normal physiological conditions, [Cl^−^]_i_ was around 30–40 mM in RECs,^40^ which was consistent with our findings. When the [Cl^−^]_i_ reached 75 mM or above, a level similar to that in cells stimulated with SARS-CoV-2 N protein, secretion of IL-1β was maximized in the bronchial epithelial cells, leading to robust inflammatory response.^41^ Previous studies revealed that SARS-CoV-2 N protein shares a 91 and 49% similarity of the protein sequence as well as secondary structure to SARS-CoV and MERS-CoV, respectively.^42^ We, therefore, attempted to investigate the effects of N proteins of these two coronaviruses on [Cl^−^]_i_ of RECs. Interestingly, the [Cl^−^]_i_ increased from 33.20 ± 5.49 mM to 83.47 ± 8.38 mM and 67.62 ± 5.45 mM after stimulation with the N proteins of SARS-CoV and MERS-CoV, respectively (Supplementary Fig. S8). These findings were consistent with the previously reported associations between SARS-CoV N protein stimulation and NF-κB-mediated inflammatory responses.^43^ The accumulation of intracellular Cl^−^ induced by coronavirus N proteins might be a phenomenon generalizable to the sarbecoviruses. On the other hand, Cl^−^ efflux reportedly induced the release of IL-1β in bone marrow-derived macrophages, polymorphonuclear leukocytes, and endothelial cells,^44^ which indicated the link between the [Cl^−^]_i_ and the secretion of pro-inflammatory cytokines, with mechanisms to be defined. Although SGK1 played crucial roles in regulating the expression of cytokines,^23^ the secretion of cytokines mediated by the Cl^−^-sensing kinase warranted further investigations. Interestingly, inhibition of Cl^−^ influx suppressed the replication of the hepatitis C virus, suggesting that the elevation of [Cl^−^]_i_ is necessary for virus replication.^45^ Additionally, the increased [Cl^−^]_i_ might also contribute to mucus retention, pulmonary edema, and impaired respiratory function after viral infections.^46^ Therefore, our findings suggested that an elevation in [Cl^−^]_i_ may not only facilitate virus replication but also trigger pulmonary inflammation following viral infections (i.e., viral pneumonia). Notably, the elevated [Cl^−^]_i_ has been observed in infections with bacteria, parasites, and viruses,^47^ implying that increased [Cl^−^]_i_ might be a universal marker for infection-induced inflammation and that intracellular Cl^−^ could serve as a pathological second messenger in disease. This might have therapeutic implications for ameliorating the inflammatory responses in other systemic disorders (i.e., cancer, cystic fibrosis, Bartter syndrome, congenital chloride diarrhea).^48^

Currently, N protein has been recognized as an early diagnostic marker for SARS-CoV-2 infection and a target for vaccine development owing to the robust T cell responses.^49,50^ Because the serum N protein antigen level was associated with the inflammatory responses and disease severity among COVID-19 patients,^51^ targeting the N protein and the downstream signaling pathways represents a new therapeutic strategy for COVID-19. Our study has revealed that inhibition of PDE4 attenuated the airway inflammation stimulated by N protein via reduction of the [Cl^−^]_i_ which approached the resting-state levels. The anti-inflammatory activities of rolipram have been consistently proven.^52^ In a pneumococcal pneumonia model, rolipram downregulated airway cytokine expression levels, and ameliorated neutrophil recruitment and lung damage.^53^ Our results, therefore, provide the basis for repurposing PDE4 inhibitors such as rolipram or roflumilast as the adjunct therapy for COVID-19 as described previously.^54^

Collectively, SARS-CoV-2 N protein triggered an elevation of [Cl^−^]_i_ via downregulating CFTR by interacting with the Smad3-miR-145 signaling pathways, which could elicit robust inflammatory responses in the respiratory epithelium. Intracellular Cl^−^ disequilibrium contributes to SARS-CoV-2-induced airway inflammation, providing novel insights into the pathogenesis of COVID-19 and the identification of the candidates for drug development.

## Materials and methods

### Cell culture and viral structural protein stimulation

Two REC cell lines—the BEAS-2B and A549 cells were obtained from Jennio Biotech (China), and, were cultured in DMEM (high glucose, Hyclone, USA). Two other REC cell lines including the 16HBE14o- and Calu-3 cells [the gifts from Prof. Wing-Hung Ko (The Chinese University of Hong Kong, Hong Kong, China)] were cultured in the MEM (Gibco, USA) and DMEM/F12 medium (Gibco). All media were supplemented with 1% (v/v) penicillin and streptomycin (Hyclone) and 10% (v/v) fetal bovine serum (FBS) (Gibco) and all cells were incubated at 37 °C in a 5% CO_2_-gassed humidified atmosphere. Before the experiments, the cells were starved of serum for 12 h. Next, the SARS-CoV-2 structural proteins including S1, S2 and N protein (50 μg/ml, Novoprotein, China), SARS-CoV N protein (50 μg/ml, Abclonal, China) or MERS-CoV N protein (50 μg/ml, Abclonal) were added. The selective inhibitors, including EMD638683 (50 μM), PDTC (100 μM), and rolipram (20 μM) (Sigma Aldrich, USA), were added at 1 h before the N protein stimulation.

### Isolation and culture of hPAECs

The hPAECs were collected via bronchial brushing from three study participants who gave written informed consent and were recruited from the outpatient clinics of the First Affiliated Hospital of Guangzhou Medical University (Guangzhou, China). The study was approved by the Ethics Committee of The First Affiliated Hospital of Guangzhou Medical University. Further details regarding the demographic characteristics are shown in Supplementary Table S1.

The cells were detached from the brush in DMEM containing 1% (v/v) penicillin and streptomycin. After centrifugation at 500×*g* for 5 min, the cells were re-suspended in PneumaCult™-Ex Plus Medium (05040, STEMCELL, Canada) containing 1% (v/v) penicillin and streptomycin and cultured at 37 °C in a 5% CO_2_-gassed humidified atmosphere. The cells were subcultured until 50–60% confluence. The characteristic of the hPAECs is shown in Supplementary Fig. S7a.

### qPCR

The RNA from the cells or lungs of the N protein-intratracheally instilled mice was extracted by using the SteadyPure RNA extraction kit (Accurate Biology, China). The RNA from the lung homogenates of SARS-CoV-2-infected mice was extracted by using TRIzol (Invitrogen, USA). The qPCR assay was performed by using EVO M-MLV RT kit (Accurate Biology) and SYBR Green (Accurate Biology) on a LightCycler 480 platform (Roche, Switzerland). The PCR was proceeded for 30 s at 95 °C, 40 cycles of 95 °C for 5 s, 58 °C for 15 s, and 72 °C for 20 s. The relative mRNA change was calibrated and normalized by using the 2^-ΔΔCt^ method. All primers (Tsingke Biotechnology, China) are shown in Supplementary Table S2.

### RNA-seq analysis

The RNA from the cells was extracted by using the SteadyPure RNA extraction kit (Accurate Biology). RNA-seq was performed by Gene Denovo Biotechnology (Guangzhou, China). The original gene expression level was normalized to the fragment per kilobase of transcript per million mapped reads. Subsequently, the DEGs between groups were identified with the cut-off of *P* < 0.05 and the absolute logarithmically transformed fold-change (|FC | ) ≥ 1. The statistical analysis of RNA-Seq was performed with the OmicShare and visualized with Hiplot.

### Western blot and co-immunoprecipitation assays

Western blot was performed as described previously with minor modifications.^55^ Briefly, the protein was extracted by using the radioimmunoprecipitation assay lysis buffer (E-BC-R327, Elabscience Biotechnology, China) containing the protease-phosphatase inhibitor cocktail (BestBio Science, China) and phenylmethylsulfonyl fluoride (1 mM). The primary antibodies including anti-IκBα (4814, 1:1000, CST, USA), anti-phospho-IκBα (2859, 1:1000, CST), anti-NF-κB p65 (59674, 1:1000, CST), anti-phospho-NF-κB p65 (3033, 1:1000, CST), anti-CFTR (ab2784, 1:1000, Abcam, UK), anti-phospho-SGK1 (36-002, 1:2000, Millipore, USA), anti-SGK1 (ab59337, 1:1000, Abcam), anti-SARS-CoV-2 N protein (33717, 1:1000, CST), anti-Smad3 (9523, 1:1000, CST) and anti-GAPDH (5174, 1:1500, CST), and a horse radish peroxidase-conjugated secondary antibody (E030120-01, 1:20000, Earthox, USA) were used in this study. The proteins were visualized by using Femto-Sig ECL chemiluminescence substrate on a chemiluminescent imaging system (Tanon, 5200, China). The bands were analyzed by using Image J (National Institutes of Health, USA).

For co-immunoprecipitation, the protein was extracted by using the lysis buffer containing 150 mM NaCl, 50 mM Tris-HCl (pH 7.4), 5 mM EDTA, 1% Nonidet-p40, and 10% glycerol. An aliquot of each whole-cell lysate sample was used to confirm the protein expression levels and the rest of the cell lysates were used for performing the co-immunoprecipitation assays. The lysates were incubated with an anti-Smad3 antibody (9523, 1:100, CST) at 4 °C overnight and mixed with 40 μl of Protein A/G PLUS-Agarose (sc-2003, Santa Cruz Biotechnology, USA) for 2 h at 4 °C. The immunoprecipitants were washed with the cold lysis buffer, boiled in 2 × sodium dodecyl sulfate (SDS) loading buffer, and subject to Western blot assays.

### GST pull-down assay

GST Resin (GE Health, USA) was washed with phosphate-buffered saline (PBS). GST protein (11213-HNAB, SinoBiological, China), human Smad3 protein (His & GST tag, 50991-M20B, SinoBiological), and SARS-CoV-2 N protein (6His tag) were added to the GST Resin either individually or in combination. Next, the GST Resins were washed with PBS after co-incubation at 4 °C for 4 h. Finally, the proteins were eluted with the eluent and detected by performing Western blot assays.

### Immunofluorescence assay

Cells were cultured in the glass Petri dishes and the paraffin section was performed on the lung tissues of mice. The cells were fixed with 4% paraformaldehyde for 15 min. The samples were permeabilized with Triton x-100 for 30 min. After washing with cold PBS, the samples were blocked with 3% bovine serum albumin (BSA) for 1 h at room temperature, and incubated with the primary antibodies including anti-CFTR (sc-376683, 1:200, Santa Cruz Biotechnology), anti-phospho-SGK1 (36-002, 1:200, Millipore), anti-SARS-CoV-2 N protein (ab271180, rabbit mAb, 1:500, Abcam), anti-SARS-CoV-2 N protein (33717, mouse mAb, 1:400, CST), anti-Smad3 (9523, 1:100, CST), anti-pan-Keratin (4545, 1:500, CST), anti-Vimentin (EPR3776, 1:500, Abcam), anti-F4/80 (D4C8V) (30325, 1:500, CST), and anti-β IV Tubulin (bs-20694R, 1:200, Bioss, China) at 4 °C overnight. The samples were washed with PBS and incubated with the fluorescence-labeled secondary antibodies including Donkey anti-Rabbit IgG (A10042, Invitrogen) and Donkey anti-Mouse IgG (A21202, Invitrogen) at room temperature for 1 h. Finally, DAPI (C0060, Solarbio, China) was added to label the cell nuclei at room temperature for 5 min. The fluorescence was recorded by using a confocal imaging system (TCS SP8 STED 3X, Leica).

### Measurement of *I*_SC_

The *I*_SC_ measurement was performed as described previously with minor modifications.^56^ Briefly, the 16HBE14o- cells were cultured in the 0.45 cm^2^ permeable filters (Millipore, USA). The *I*_SC_ was recorded by using a voltage–current clamp amplifier (VCC MC6, Physiologic instrument, USA) and a biosignal collection system (BL-420E+, Chengdu Technology, China). The change in *I*_SC_ was defined as the value at 600 s after adding forskolin (10 μM) and was normalized to the vehicle control group.

### Intratracheal instillation and virus infection in mice

The ICR mice (male, 6 weeks old) were purchased from the Laboratory Animal Center of Sun Yat-sen University (Guangzhou, China). The mice model of SARS-CoV-2 N protein stimulation was established as described previously with a modified procedure.^57^ Briefly, the mice were anesthetized with tribromoethyl alcohol [20 μl 1.25% (w/v) saline solution per gram of the body weight] intraperitoneally. Next, the mice were subject to intratracheal instillation of PBS or SARS-CoV-2 N protein (0.25 or 0.5 mg/kg) (DRA31, NovoProtein, China). Because the augmented IκB phosphorylation did not differ significantly in the lungs isolated from mice which were stimulated with 0.25 or 0.5 mg/kg N protein (Supplementary Fig. S2c), we adopted the dose of 0.25 mg/kg in the main experiments. For the inhibitor treatment group, mice were intraperitoneally injected with EMD638683 (10 mg/kg) (HY-15193, MedChemExpress, USA), PDTC (100 mg/kg) (Sigma Aldrich), or rolipram (10 mg/kg) (Sigma Aldrich) before N protein stimulation. After stimulation for 24 h, the mice were euthanatized and the subsequent experiments were performed. All experiments were approved by the Institutional Animal Care and Use Committee (IACUC), Sun Yat-sen University (Guangzhou, China; Approval No: SYSU-IACUC-2020-B0013).

The SARS-CoV-2 challenge murine model was constructed as described previously.^58^ Briefly, BALB/c mice were purchased from the Hunan SJA Laboratory Animal Co. (China). Mice were anesthetized and intranasally instilled with Ad5-ACE2 (2.5 × 10^8^ FFU) in a total volume of 75 μL DMEM. Five days after transduction, mice were intranasally instilled 1 × 10^5^ plaques forming units (PFU) SARS-CoV-2 which was dissolved in 50 μL DMEM. The lungs used for qPCR assay were isolated from SARS-CoV-2-challenged mice at day 2 or day 4 post infection. The lungs used for histologic or immunofluorescence assay were isolated from SARS-CoV-2-challenged mice at day 4 post infection. All works related to SARS-CoV-2 were carried out in the Biosafety Level 3 Laboratory of Guangzhou Customs Technology Center (Guangzhou, China). All procedures were approved by the Institutional Animal Care and Use Committees of the Guangzhou Medical University (Guangzhou, China; Approval No: 202068).

### Measurement of intracellular Cl^−^

The measurement of [Cl^−^]_i_ was performed as described previously.^59^ Briefly, the cells were seeded on a glass cover slip. The mPAECs were obtained from the mouse trachea by adding 0.25% (w/v) trypsin (Gibco) at 4 °C for 16 h. N-(ethoxycarbonylmethyl)-6-methoxyquinolinium bromide (MQAE, 5 mM, Invitrogen) was used to document changes in [Cl^−^]_i_. The nigericin and tributyltin-contained buffer was used to equilibrate the intracellular and extracellular concentration of Cl^−^ and gluconate was used to replace the Cl^−^ at different concentrations. The fluorescence was recorded at 350 nm by using an imaging system (IX83, Olympus, Japan).

### Construction of the CRISPR-Cas9-based *SGK1* KO cells

The *SGK1* KO BEAS-2B cells were constructed by HYY Medical Science (Guangzhou, China) using the PXC9-puro plasmid with either a single guide RNA (sgRNA) targeting the SGK1 gene or a scrambled sgRNA with no target as described previously.^23^ The sequences of the sgRNA are shown in Supplementary Table S3.

### Intracellular cAMP measurement

The content of the intracellular cAMP in the BEAS-2B cell lysates was detected by using the cAMP enzyme immunoassay kit (KGE002B, R&D Systems, USA) according to the manufacturer’s instructions. Bicinchoninic acid Protein Assay Kit (PC0020, Solarbio, China) was used to measure the protein content of the cell lysates.

### Histologic assay

The mouse lung tissues were isolated and fixed in 4% paraformaldehyde (Solarbio, China) for more than 24 h and embedded in paraffin. For histopathological analysis, 4 μm sections were stained with the standard hematoxylin & eosin (H&E) solutions. Finally, the H&E staining images were captured by using an imaging system (ECLIPSE 50i, Nikon, Japan).

### Statistical analysis

Data were all shown as mean ± SD with distinct dots for each measurement. Unpaired two-tailed *t*-test analysis was used to compare the differences between the two groups. One-way ANOVA followed by Dunnett multiple comparison test was used for comparing three or more groups. *P* value of less than 0.05 was considered as statistically significant. Statistical analysis was analyzed by using GraphPad Prism 8.3.0 (GraphPad Software, USA).

## Supplementary information

Supplementary Materials

## Data Availability

All data supporting the findings of this research are available within the article and its supplementary information or from the corresponding author upon reasonable request.
