# Supplementary material for: SARS-CoV-2 nucleocapsid protein triggers hyperinflammation via protein-protein interaction-mediated intracellular Cl− accumulation in respiratory epithelium
Source: Signal Transduct Target Ther. 2022 Jul 27;7:255. doi: 10.1038/s41392-022-01048-1 (PMC9328007; doi:10.1038/s41392-022-01048-1)
Supplement: Supplementary file 1 — Supplementary Materials [file 41392_2022_1048_MOESM1_ESM.docx]

**Supplementary Materials for**

**SARS-CoV-2 nucleocapsid protein triggers hyperinflammation via protein-protein interaction-mediated intracellular Cl^−^ accumulation in respiratory epithelium**

Lei Chen^#^, Wei-Jie Guan^#^, Zhuo-Er Qiu^#^, Jian-Bang Xu^#^, Xu Bai, Xiao-Chun Hou, Jing Sun, Su Qu, Ze-Xin Huang, Tian-Lun Lei, Zi-Yang Huang, Jincun Zhao, Yun-Xin Zhu, Ke-Nan Ye, Zhao-Rong Lun, Wen-Liang Zhou^*^, Nan-Shan Zhong^*^, Yi-Lin Zhang^*^

**Correspondence to:** [zhangylin9@mail.sysu.edu.cn](mailto:zhangylin9@mail.sysu.edu.cn) (Y-LZ); [lsszwl@mail.sysu.edu.cn](mailto:lsszwl@mail.sysu.edu.cn) (W-LZ); [nanshan@vip.163.com](mailto:nanshan@vip.163.com) (N-SZ)

**This PDF file includes:**

Figures S1 to S8

Tables S1 to S3

**Supplementary Figures**

**
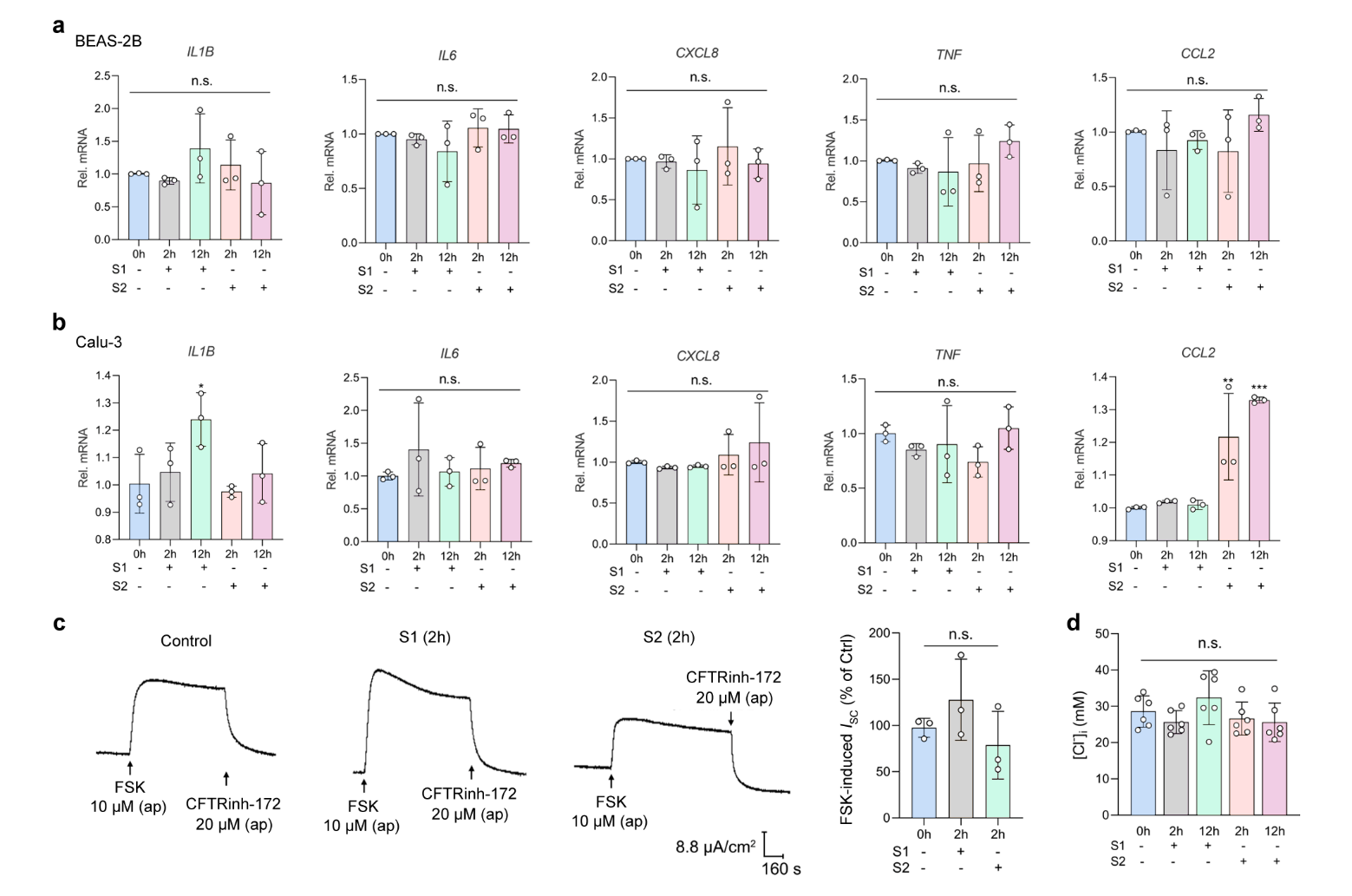
Figure. S1.**

**SARS-CoV-2 S1 and S2 protein stimulation did not affect the mRNA expression of pro-inflammatory cytokines, transepithelial Cl^−^ secretion, and [Cl^−^]_i_ in RECs. (a, b)** The mRNA expression levels of pro-inflammatory cytokines after stimulation with S1 and S2 protein (50 μg/ml) in BEAS-2B and Calu-3 cells (*n* = 3). **(c)** Representative traces showing the effect of S1 and S2 protein (50 μg/ml) on *I*_SC_ responses induced by forskolin (FSK, 10 μM) in 16HBE14o- cells, with the statistical analysis (*n* = 3). **(d)** The changes in [Cl^−^]_i_ after S1 and S2 protein (50 μg/ml) stimulation in BEAS-2B cells (*n* = 6 cells). Data are shown as mean ± SD, ^*^ *P* < 0.05, ^**^ *P* < 0.01, ^***^ *P* < 0.001 compared with the control group or indicated by lines, n.s., *P* > 0.05.

**Figure. S2.**


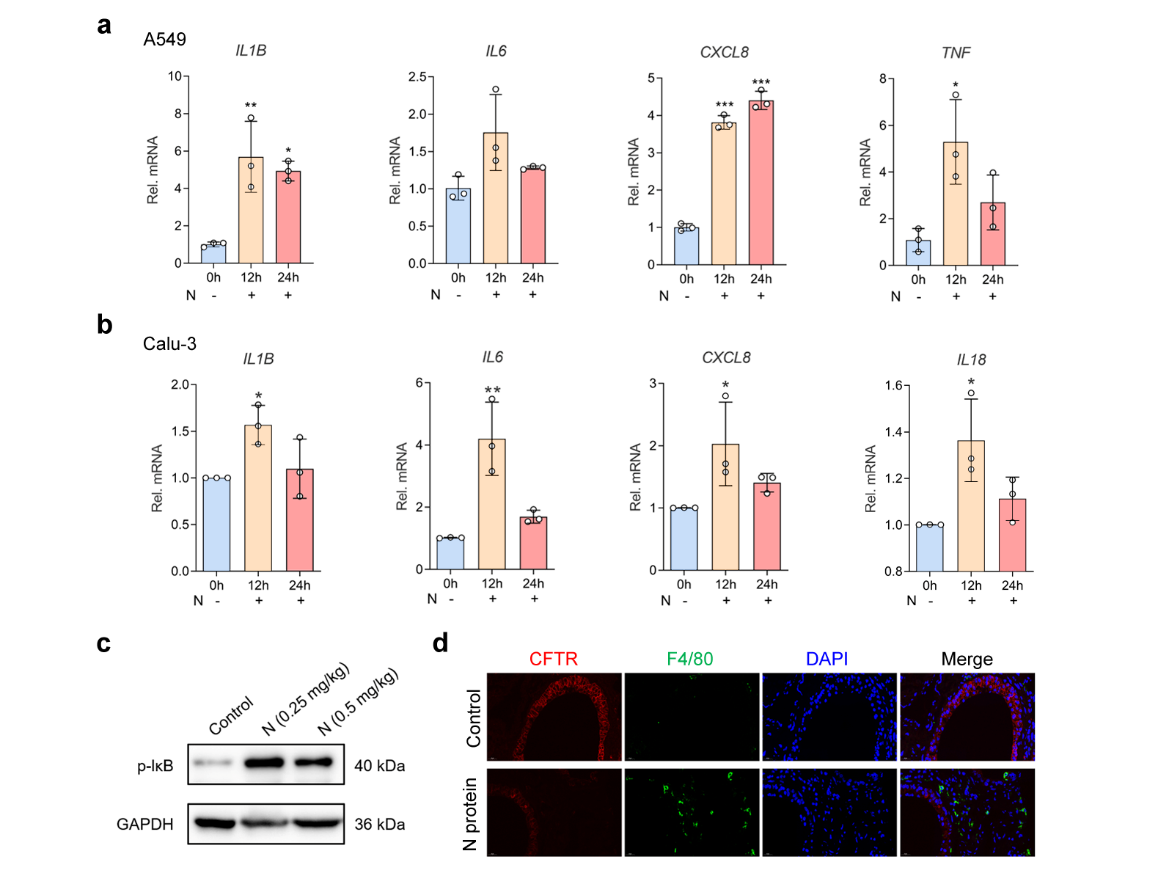


**SARS-CoV-2 N protein elicited inflammatory responses and down-regulation of CFTR in RECs. (a, b)** The mRNA expression levels of pro-inflammatory cytokines after stimulation with N protein (50 μg/ml) in A549 or Calu-3 cells (*n* = 3). Data are shown as mean ± SD, ^*^ *P* < 0.05, ^**^ *P* < 0.01, ^***^ *P* < 0.001 compared with the control group. **(c)** The phosphorylation level of IκB after N protein (0.25 or 0.5 mg/kg) stimulation in mice. **(d)** Fluorescence labeling of CFTR (red) and F4/80 (green) in mouse lung slices with DAPI-labeled nuclei (blue) after N protein (0.25 mg/kg) stimulation, revealing that the CFTR-expressing cells had the morphology of pseudostratified columnar epithelium lining the conducting airways, which was not co-localized with F4/80, a marker of macrophages. Scale bars, 20 μm.

**Figure. S3.**


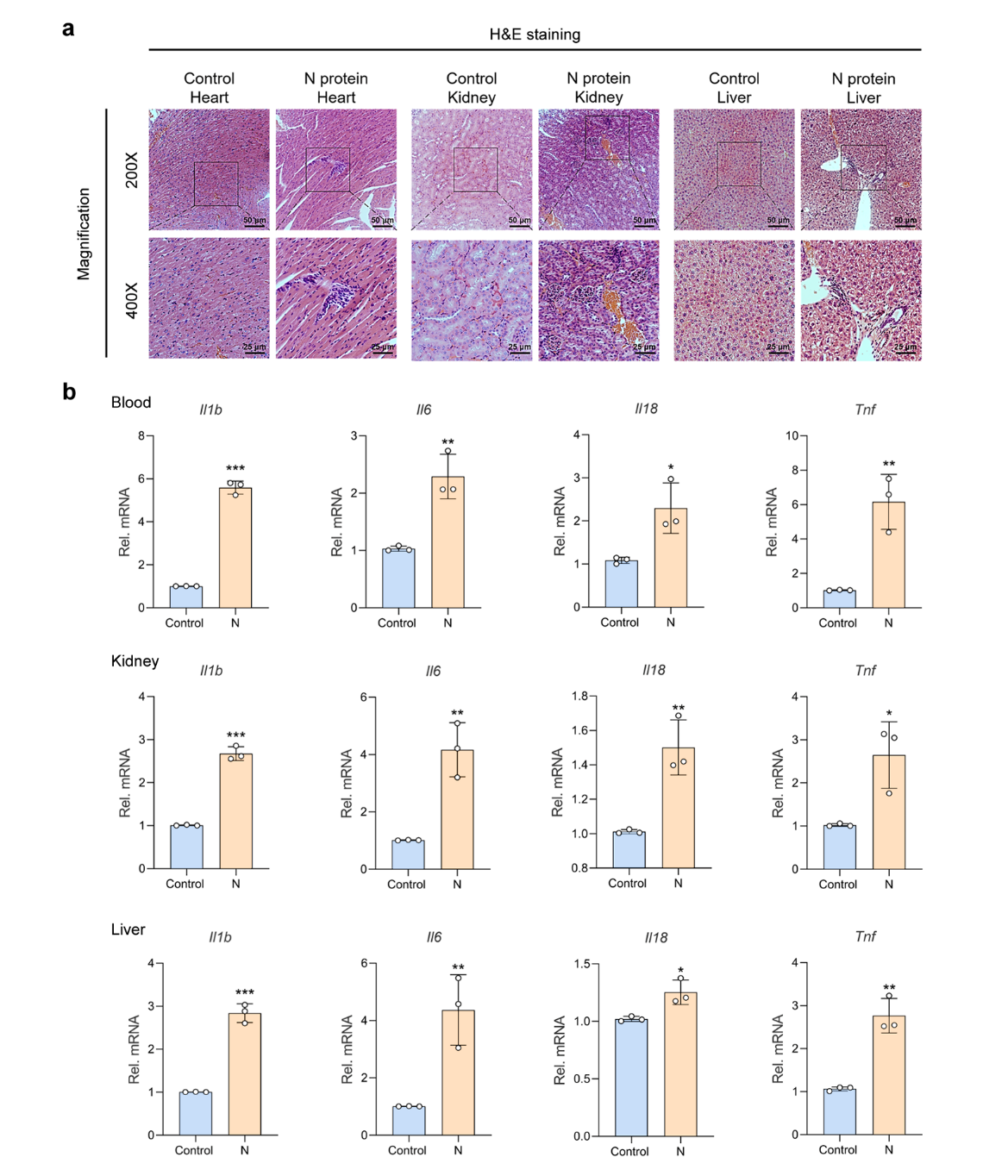


**Intratracheal instillation of SARS-CoV-2 N protein elicited systemic inflammation in mice.** (a) H&E staining of main organs from N protein (0.25 mg/kg)-stimulated mice. Scale bars, 50 μm and 25 μm. (b) The mRNA expression levels of pro-inflammatory cytokines in mice stimulated by N protein (0.25 mg/kg) (*n* = 3). Data are shown as mean ± SD, ^*^ *P* < 0.05, ^**^ *P* < 0.01, ^***^ *P* < 0.001 compared with the control group.

**Figure. S4.**


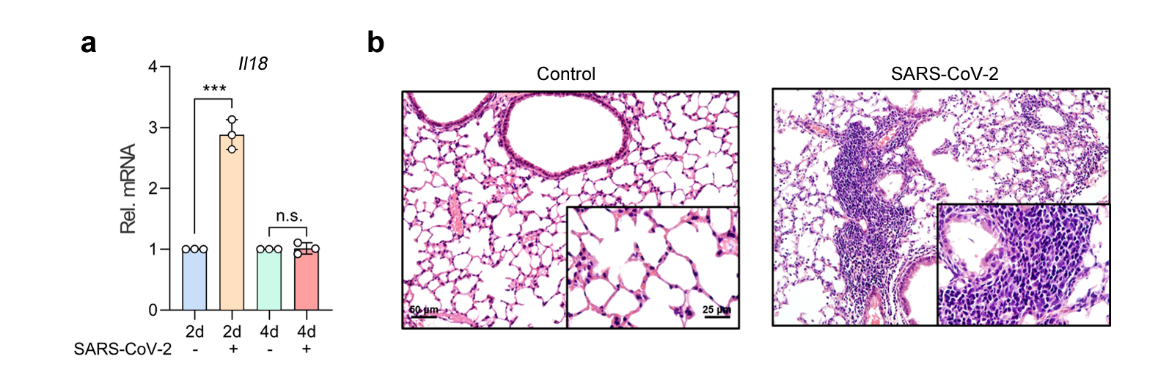


**SARS-CoV-2 infection developed pneumonia in hACE2-transduced mice. (a)** The mRNA expression level of *Il18* in SARS-CoV-2 (1×10^5^ PFU)-infected mice (*n* = 3). Data are shown as mean ± SD, ^***^ *P* < 0.001, n.s., *P* > 0.05. **(b)** H&E staining of lung slices from the control and SARS-CoV-2 (1×10^5^ PFU)-infected mice. Scale bars, 50 μm and 25 μm.

**Figure. S5.**

**
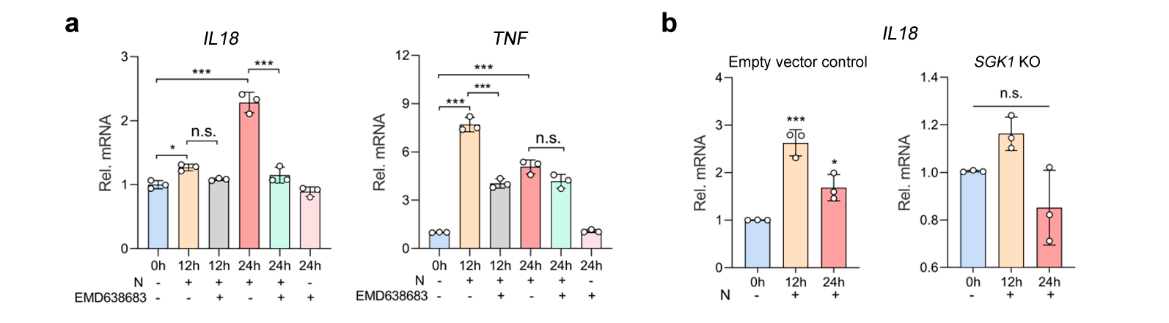
**

**Involvement of SGK1 in SARS-CoV-2 N protein-induced inflammatory responses in RECs. (a)** The effect of EMD638683 (50 μM) on the mRNA expressions of pro-inflammatory cytokines in N protein (50 μg/ml)-stimulated BEAS-2B cells (*n* = 3). **(b)** The mRNA expressions of *IL18* after N protein (50 μg/ml) stimulation in the empty vector control or *SGK1* KO BEAS-2B cells (*n* = 3). Data are shown as mean ± SD, ^*^ *P* < 0.05, ^***^ *P* < 0.001 compared with the control group or indicated by lines, n.s., *P* > 0.05.

**Figure. S6.**

**
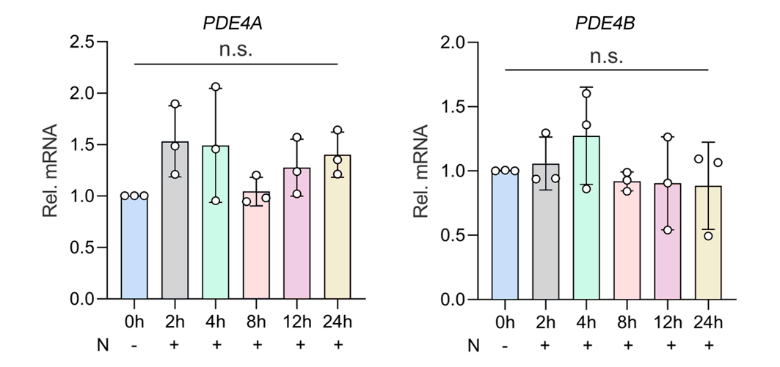
**

**The effect of SARS-CoV-2 N protein stimulation on *PDE4* mRNA expression in RECs.** The mRNA expression levels of *PDE4A* and *PDE4B* after stimulation with N protein (50 μg/ml) in BEAS-2B cells (*n* = 3). Data are shown as mean ± SD, n.s., *P* > 0.05.

**
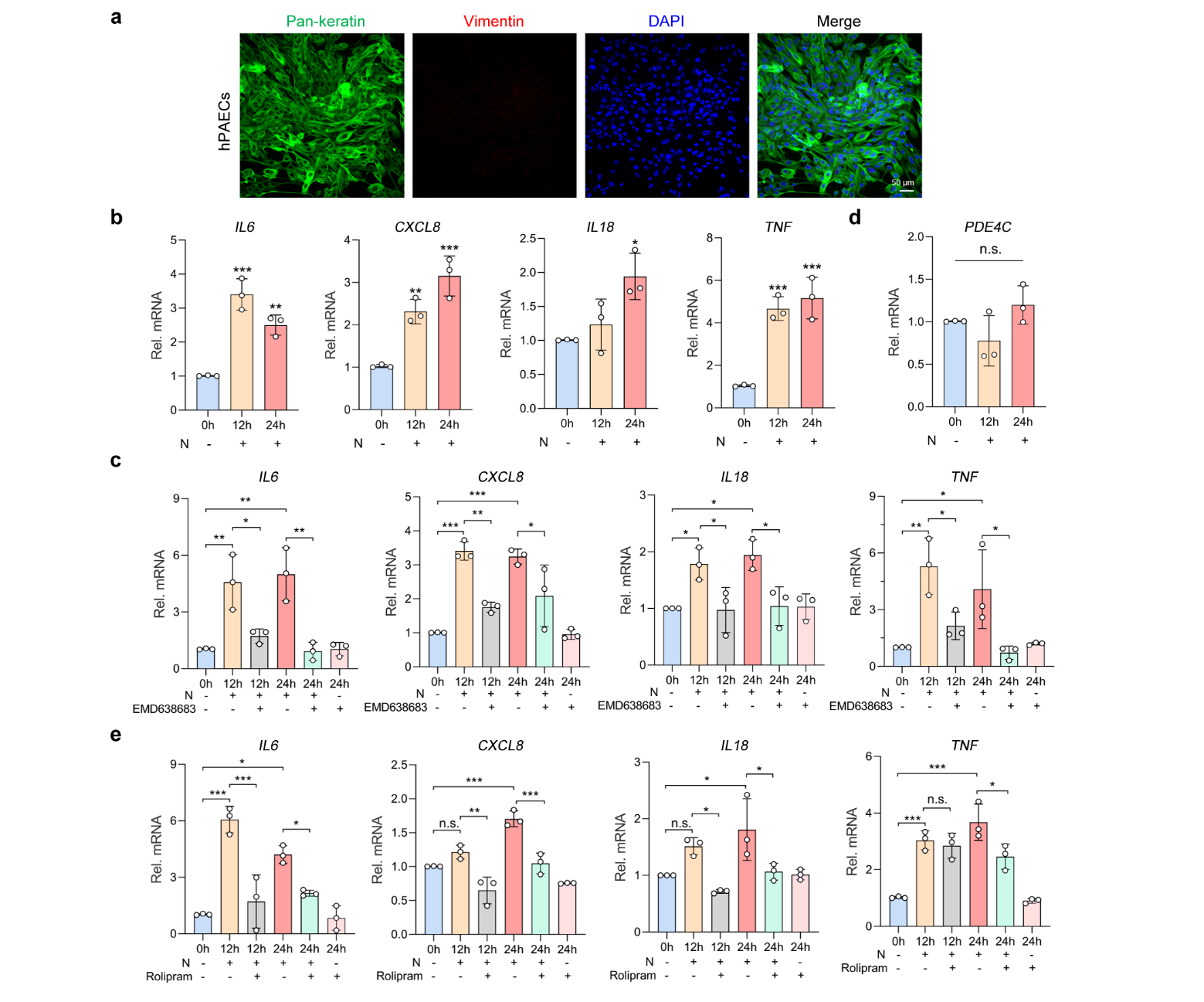
Figure. S7.**

**Characteristic of the hPAECs and the anti-inflammatory effect of EMD638683 and rolipram in SARS-CoV-2 N protein-stimulated hPAECs. (a)** Fluorescence labeling of pan-keratin (green), a marker of epithelial cells, and vimentin (red), a marker of fibroblasts, in hPAECs with DAPI-labeled nuclei (blue). Scale bars, 50 μm. **(b)** The mRNA expression levels of cytokines in N protein (50 μg/ml)-stimulated hPAECs (*n* = 3). **(c, e)** The effects of **(c)** EMD638683 (50 μM) or **(e)** rolipram (20 μM) on the mRNA expressions of cytokines in N protein (50 μg/ml)-stimulated hPAECs (*n* = 3). **(d)** The mRNA expressions of *PDE4C* in the N protein (50 μg/ml)-stimulated hPAECs (*n* = 3). Data are shown as mean ± SD, ^*^ *P* < 0.05, ^**^ *P* < 0.01, ^***^ *P* < 0.001 compared with the control group or indicated by lines, n.s. *P* > 0.05.

**Figure. S8.**


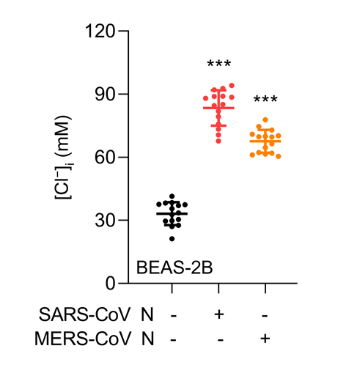


**The effects of** **SARS-CoV and MERS-CoV N protein on [Cl^−^]_i_ in RECs.** The changes in [Cl^−^]_i_ after SARS-CoV and MERS-CoV N protein (50 μg/ml) stimulation for 12 h in BEAS-2B cells (*n* = 15 cells). Data are shown as mean ± SD, ^***^ *P* < 0.001 compared with the control group.

**Supplementary Table**

**Table S1.** Age, gender, and the location of bronchial biopsy in study participants

|  | | **Study participants characteristics** |
| --- | --- | --- |
| Age (years) | | 53.0 ± 8.4 |
| Gender | Male | 1 |
|  | Female | 2 |
| Location for bronchial mucosal biopsy | | Left principal bronchus |
|  |  | Left principal bronchus |
|  |  | Left principal bronchus |

**Table S2.** Primers used in this research

| Primer name | Sequence |
| --- | --- |
| *IL1B* Forward Primer  *IL1B* Reverse Primer  *IL6* Forward Primer  *IL6* Reverse Primer  *CXCL8* Forward Primer  *CXCL8* Reverse Primer  *IL18* Forward Primer  *IL18* Reverse Primer  *TNF* Forward Primer  *TNF* Reverse Primer  *GAPDH* Forward Primer  *GAPDH* Reverse Primer  *CFTR* Forward Primer  *CFTR* Reverse Primer  *PDE4A* Forward Primer  *PDE4A* Reverse Primer  *PDE4B* Forward Primer  *PDE4B* Reverse Primer  *PDE4C* Forward Primer  *PDE4C* Reverse Primer  *PDE4D* Forward Primer  *PDE4D* Reverse Primer  *Il1b* Forward Primer  *Il1b* Reverse Primer  *Il6* Forward Primer  *Il6* Reverse Primer  *Il18* Forward Primer  *Il18* Reverse Primer  *Tnf* Forward Primer  *Tnf* Reverse Primer  *Cftr* Forward Primer  *Cftr* Reverse Primer  *Hprt* Forward Primer  *Hprt* Reverse Primer  *Pde4a* Forward Primer  *Pde4a* Reverse Primer  *Pde4b* Forward Primer  *Pde4b* Reverse Primer  *Pde4c* Forward Primer  *Pde4c* Reverse Primer  *Pde4d* Forward Primer  *Pde4d* Reverse Primer | TTCGACACATGGGATAACGAGG  TTTTTGCTGTGAGTCCCGGAG  CCTGAACCTTCCAAAGATGGC  TTCACCAGGCAAGTCTCCTCA  ACTGAGAGTGATTGAGAGTGGAC  AACCCTCTGCACCCAGTTTTC  TCTTCATTGACCAAGGAAATCGG  TCCGGGGTGCATTATCTCTAC  GAGGCCAAGCCCTGGTATG  CGGGCCGATTGATCTCAGC  TGCACCACCAACTGCTTAGC  GGATGCAGGGATGATGTTCT  TGCCCTTCGGCGATGTTT  GCGATAGAGCGTTCCTCCTTG  GGGGTGAAGACCGATCAAGAA  CGACACGCAAAAGATGTTCAG  CGGCTGGGAGAGAGGGTTATT  TCTCCAGAGGTCGATCCCAAG  GAGACGCTAGACGAGCTGGA  GTGGGTCAACTCCCGGTTC  TGTGTGACAAGCACAATGCTTCC  CACGATTGTCCTCCAAAGTGTCC  AACCTGCTGGTGTGTGACGTTC  CAGCACGAGGCTTTTTTGTTGT  AGTTGCCTTCTTGGGACTGATG  CAGGTCTGTTGGGAGTGGTATC  GACAGCCTGTGTTCGAGGATATG  TGTTCTTACAGGAGAGGGTAGAC  GACGTGGAAGTGGCAGAAGAG  TGCCACAAGCAGGAATGAGA  CTGGACCACACCAATTTTGAGG  GCGTGGATAAGCTGGGGAT  TCAGTCAACGGGGGACATAAA  GGGGCTGTACTGCTTAACCAG  AATGCCCTACAGACGCCTG  GACGGTGTTGGCCCATTTT  GACTACATTGTCCATCCACTGTG  CTTGAGCATCCGGTTGAACCA  TCCGAGAGCCAGTGGATTCT  CCTTGAGTTCCAATCGTGAAGAC  TTTTGCCAGTGCAATACATGATG  CAGAGCGAGTTCCGAGTTTGT |

**Table S3.** The sequences of the sgRNA used in this research

| sgRNA name | Sequence |
| --- | --- |
| sgRNA targeting SGK1  scrambled gRNA | GCACATTGCAGGTACGAAGG  GTAGAGAGCGGCGCGCCTAC |
